# Supplementary material for: Sensorimotor Rhythm-Based Brain–Computer Interfaces for Motor Tasks Used in Hand Upper Extremity Rehabilitation after Stroke: A Systematic Review
Source: Brain Sci. 2022 Dec 28;13(1):56. doi: 10.3390/brainsci13010056 (PMC9856697; doi:10.3390/brainsci13010056)
Supplement: Supplementary file 1 [file brainsci-13-00056-s001.zip › brainsci-2070596-supplementary.pdf]

SEARCH STRATEGY

Pubmed

(((((("Stroke"[Mesh]) OR ((((((((((((((((((((((((((((Strokes[Title/Abstract]) OR (Cerebrovascular Accident[Title/Abstract])) OR (Cerebrovascular Accidents[Title/Abstract])) OR (CVA (Cerebrovascular Accident)[Title/Abstract])) OR (CVAs (Cerebrovascular Accident)[Title/Abstract])) OR (Cerebrovascular Apoplexy[Title/Abstract])) OR (Apoplexy, Cerebrovascular[Title/Abstract])) OR (Vascular Accident, Brain[Title/Abstract])) OR (Brain Vascular Accident[Title/Abstract])) OR (Brain Vascular Accidents[Title/Abstract])) OR (Vascular Accidents, Brain[Title/Abstract])) OR (Cerebrovascular Stroke[Title/Abstract])) OR (Cerebrovascular Strokes[Title/Abstract])) OR (Stroke, Cerebrovascular[Title/Abstract])) OR (Strokes, Cerebrovascular[Title/Abstract])) OR (Apoplexy[Title/Abstract])) OR (Cerebral Stroke[Title/Abstract])) OR (Cerebral Strokes[Title/Abstract])) OR (Stroke, Cerebral[Title/Abstract])) OR (Strokes, Cerebral[Title/Abstract])) OR (Stroke, Acute[Title/Abstract])) OR (Acute Stroke[Title/Abstract])) OR (Acute Strokes[Title/Abstract])) OR (Strokes, Acute[Title/Abstract])) OR (Cerebrovascular Accident, Acute[Title/Abstract])) OR (Acute Cerebrovascular Accident[Title/Abstract])) OR (Acute Cerebrovascular Accidents[Title/Abstract])) OR (Cerebrovascular Accidents, Acute[Title/Abstract]))) OR ("Brain Ischemia"[Mesh])) OR (((((((((Brain Ischemias[Title/Abstract]) OR (Ischemia, Brain[Title/Abstract])) OR (Ischemic Encephalopathy[Title/Abstract])) OR (Encephalopathy, Ischemic[Title/Abstract])) OR (Ischemic Encephalopathies[Title/Abstract])) OR (Cerebral Ischemia[Title/Abstract])) OR (Cerebral Ischemias[Title/Abstract])) OR (Ischemias, Cerebral[Title/Abstract])) OR (Ischemia, Cerebral[Title/Abstract]))) OR (Cerebral Hemorrhage[MeSH Terms])) OR (((((((((((((((((((((((Hemorrhage, Cerebrum[Title/Abstract]) OR (Cerebrum Hemorrhage[Title/Abstract])) OR (Cerebrum Hemorrhages[Title/Abstract])) OR (Hemorrhages, Cerebrum[Title/Abstract])) OR (Cerebral Parenchymal Hemorrhage[Title/Abstract])) OR (Cerebral Parenchymal Hemorrhages[Title/Abstract])) OR (Hemorrhage, Cerebral Parenchymal[Title/Abstract])) OR (Hemorrhages, Cerebral Parenchymal[Title/Abstract])) OR (Parenchymal Hemorrhage, Cerebral[Title/Abstract])) OR (Parenchymal Hemorrhages, Cerebral[Title/Abstract])) OR (Intracerebral Hemorrhage[Title/Abstract])) OR (Hemorrhage, Intracerebral[Title/Abstract])) OR (Hemorrhages, Intracerebral[Title/Abstract])) OR (Intracerebral Hemorrhages[Title/Abstract])) OR (Hemorrhage, Cerebral[Title/Abstract])) OR (Cerebral Hemorrhages[Title/Abstract])) OR (Hemorrhages, Cerebral[Title/Abstract])) OR (Brain Hemorrhage, Cerebral[Title/Abstract])) OR (Brain Hemorrhages, Cerebral[Title/Abstract])) OR (Cerebral Brain Hemorrhage[Title/Abstract])) OR (Cerebral Brain Hemorrhages[Title/Abstract])) OR (Hemorrhage, Cerebral Brain[Title/Abstract])) OR (Hemorrhages, Cerebral Brain[Title/Abstract]))) AND (("Brain-Computer Interfaces"[Mesh]) OR (((((((((((((((Brain Computer Interfaces[Title/Abstract]) OR (Interface, Brain-Computer[Title/Abstract])) OR (Interfaces, Brain-Computer[Title/Abstract])) OR (Brain-Computer Interface[Title/Abstract])) OR (Brain Computer Interface[Title/Abstract])) OR (Brain-Machine Interfaces[Title/Abstract])) OR (Brain-Machine Interface[Title/Abstract])) OR (Interface, Brain-Machine[Title/Abstract])) OR (Interfaces, Brain-Machine[Title/Abstract])) OR (Brain Machine Interface[Title/Abstract])) OR (Brain Machine Interfaces[Title/Abstract])) OR (Interface, Brain Machine[Title/Abstract])) OR (Interfaces, Brain Machine[Title/Abstract])) OR (Machine Interface, Brain[Title/Abstract])) OR (Machine Interfaces, Brain[Title/Abstract])))

Web of science

TS=（Brain-Computer Interfaces OR Brain Computer Interfaces OR Brain-Computer Interface OR Brain Computer Interface OR Brain-Machine Interfaces OR Brain-Machine Interface OR Brain Machine Interface OR Brain Machine Interfaces）

TS=(Electroencephalography OR EEG OR Electroencephalogram OR Electroencephalograms)

TS=(Stroke* OR "Brain Infarction" OR "Cerebral Infarction" OR "Brain Ischemia" OR "Intracranial Embolism and Thrombosis" OR "Intracranial Hemorrhages" OR "Cerebrovascular Accident" OR "Cerebrovascular Accidents" OR "Cerebrovascular Apoplexy" OR "Brain Vascular Accident" OR "Brain Vascular Accidents" OR "Acute Cerebrovascular Accidents" OR Apoplexy OR "Brain Infarctions" OR "Brain Infarct" OR "Brain Infarcts" OR "Anterior Circulation Brain Infarction" OR "Anterior Cerebral Circulation Infarction" OR "Posterior Circulation Brain Infarction" OR "Cerebral Infarctions" OR "Cerebral Infarct" OR "Cerebral Infarcts" OR "Subcortical Infarction" OR "Subcortical Infarctions" OR "Posterior Choroidal Artery Infarction" OR "Anterior Choroidal Artery Infarction" OR "Brain Ischemias" OR "Ischemic Encephalopathy" OR "Ischemic Encephalopathy" OR "Ischemic Encephalopathies" OR "Cerebral Ischemia" OR "Cerebral Ischemi*" OR "Cerebral Embolism" OR "Brain Embolism" OR "Intracranial Hemorrhage" OR "Posterior Fossa Hemorrhage" OR "Posterior Fossa Hemorrhages" OR "Brain Hemorrhage" OR "Brain Hemorrhages" OR "cerebral hemorrhage" OR "hematencephalon" OR "encephalorrhagia")

ALL=("randomized controlled trial" OR "controlled clinical trial" OR "clinical trial" OR "randomized" OR clinical trial OR "placebo" OR "randomly" OR "cross-over studies")

Scopus

TITLE-ABS-KEY (brain-computer AND interfaces OR brain AND computer AND interfaces OR brain-computer AND interface OR brain AND computer AND interface OR brain-machine AND interfaces OR brain-machine AND interface OR brain AND machine AND interface OR brain AND machine AND interfaces) AND TITLE-ABS-KEY (electroencephalography OR eeg OR electroencephalogram OR electroencephalograms) AND TITLE-ABS-KEY (stroke* OR "Brain Infarction" OR "Cerebral Infarction" OR "Brain Ischemia" OR "Intracranial Embolism and Thrombosis" OR "Intracranial Hemorrhages" OR "Cerebrovascular Accident" OR "Cerebrovascular Accidents" OR "Cerebrovascular Apoplexy" OR "Brain Vascular Accident" OR "Brain Vascular Accidents" OR "Acute Cerebrovascular Accidents" OR apoplexy OR "Brain Infarctions" OR "Brain Infarct" OR "Brain Infarcts" OR "Anterior Circulation Brain Infarction" OR "Anterior Cerebral Circulation Infarction" OR "Posterior Circulation Brain Infarction" OR "Cerebral Infarctions" OR "Cerebral Infarct" OR "Cerebral Infarcts" OR "Subcortical Infarction" OR "Subcortical Infarctions" OR "Posterior Choroidal Artery Infarction" OR "Anterior Choroidal Artery Infarction" OR "Brain Ischemias" OR "Ischemic Encephalopathy" OR "Ischemic Encephalopathy" OR "Ischemic Encephalopathies" OR "Cerebral Ischemia" OR "Cerebral Ischemi*" OR "Cerebral Embolism" OR "Brain Embolism" OR "Intracranial Hemorrhage" OR "Posterior Fossa Hemorrhage" OR "Posterior Fossa Hemorrhages" OR "Brain Hemorrhage" OR "Brain Hemorrhages" OR "cerebral hemorrhage" OR

"hematencephalon" OR "encephalorrhagia") AND ALL ("randomized controlled trial" OR "controlled clinical trial" OR "clinical trial" OR "randomized" OR clinical AND trial OR "placebo" OR "randomly" OR "cross-over studies")

EMBASE

Search History (11) View

# ▲	Searches	Results	Type	Actions	Annotations
1	exp brain computer interface/	6196	Advanced	Display Results | More	
2	exp electroencephalography/	116870	Advanced	Display Results | More	
3	exp cerebrovascular accident/	219660	Advanced	Display Results | More	
4	('Brain-Computer Interfaces' or 'Brain Computer Interfaces' or 'Brain-Computer Interface' or 'Brain-Machine Interfaces' or 'Brain-Machine Interface' or 'Brain Machine Interface' or 'Brain Machine Interfaces').mp. [mp=title, abstract, heading word, drug trade name, original title, device manufacturer, drug manufacturer, device trade name, keyword, floating subheading word, candidate term word]	8600	Advanced	Display Results | More	
5	(Electroencephalography or EEG or Electroencephalogram or Electroencephalograms).mp. [mp=title, abstract, heading word, drug trade name, original title, device manufacturer, drug manufacturer, device trade name, keyword, floating subheading word, candidate term word]	221563	Advanced	Display Results | More	
6	(Stroke* or "Brain Infarction" or "Cerebral Infarction" or "Brain Ischemia" or "Intracranial Embolism and Thrombosis" or "Intracranial Hemorrhages" or "Cerebrovascular Accident" or "Cerebrovascular Accidents" or "Cerebrovascular Apoplexy" or "Brain Vascular Accident" or "Brain Vascular Accidents" or "Acute Cerebrovascular Accidents" or Apoplexy or "Brain Infarctions" or "Brain Infarct" or "Brain Infarcts" or "Anterior Circulation Brain Infarction" or "Anterior Cerebral Circulation Infarction" or "Posterior Circulation Brain Infarction" or "Cerebral Infarctions" or "Cerebral Infarct" or "Cerebral Infarcts" or "Subcortical Infarction" or "Subcortical Infarctions" or "Posterior Choroidal Artery Infarction" or "Anterior Choroidal Artery Infarction" or "Brain Ischemias" or "Ischemic Encephalopathy" or "Ischemic Encephalopathy" or "Ischemic Encephalopathies" or "Cerebral Ischemia" or "Cerebral Ischemi*" or "Cerebral Embolism" or "Brain Embolism" or "Intracranial Hemorrhage" or "Posterior Fossa Hemorrhage" or "Posterior Fossa Hemorrhages" or "Brain Hemorrhage" or "Brain Hemorrhages" or "cerebral hemorrhage" or "hematencephalon" or "encephalorrhagia").mp. [mp=title, abstract, heading word, drug trade name, original title, device manufacturer, drug manufacturer, device trade name, keyword, floating subheading word, candidate term word]	685161	Advanced	Display Results | More	
7	("randomized controlled trial" or "controlled clinical trial" or "clinical trial" or "randomized" or clinical trial or "placebo" or "randomly" or "cross-over studies").mp. [mp=title, abstract, heading word, drug trade name, original title, device manufacturer, drug manufacturer, device trade name, keyword, floating subheading word, candidate term word]	2553505	Advanced	Display Results | More	
8	1 or 4	8600	Advanced	Display Results | More	
9	2 or 5	236599	Advanced	Display Results | More	
10	3 or 6	685161	Advanced	Display Results | More	
11	7 and 8 and 9 and 10	77	Advanced	Display Results | More	

1 exp brain computer interface/

2 exp electroencephalography/

3 exp cerebrovascular accident/

4 ('Brain-Computer Interfaces' or 'Brain Computer Interfaces' or 'Brain-Computer Interface' or 'Brain-Machine Interfaces' or 'Brain-Machine Interface' or 'Brain Machine Interface' or 'Brain Machine Interfaces').mp. [mp=title, abstract, heading word, drug trade name, original title, device manufacturer, drug manufacturer, device trade name, keyword, floating subheading word, candidate term word]

5 (Electroencephalography or EEG or Electroencephalogram or Electroencephalograms).mp. [mp=title, abstract, heading word, drug trade name, original title, device manufacturer, drug manufacturer, device trade name, keyword, floating subheading word, candidate term word]

6 (Stroke* or "Brain Infarction" or "Cerebral Infarction" or "Brain Ischemia" or "Intracranial Embolism and Thrombosis" or "Intracranial Hemorrhages" or "Cerebrovascular Accident" or "Cerebrovascular Accidents" or "Cerebrovascular Apoplexy" or "Brain Vascular Accident" or "Brain Vascular Accidents" or "Acute Cerebrovascular Accidents" or Apoplexy or "Brain Infarctions" or "Brain Infarct" or "Brain Infarcts" or "Anterior Circulation Brain Infarction" or "Anterior Cerebral Circulation Infarction" or "Posterior Circulation Brain Infarction" or "Cerebral Infarctions" or "Cerebral Infarct" or "Cerebral Infarcts" or "Subcortical Infarction" or "Subcortical

Infarctions" or "Posterior Choroidal Artery Infarction" or "Anterior Choroidal Artery Infarction" or "Brain Ischemias" or "Ischemic Encephalopathy" or "Ischemic Encephalopathy" or "Ischemic Encephalopathies" or "Cerebral Ischemia" or "Cerebral Ischemi*" or "Cerebral Embolism" or "Brain Embolism" or "Intracranial Hemorrhage" or "Posterior Fossa Hemorrhage" or "Posterior Fossa Hemorrhages" or "Brain Hemorrhage" or "Brain Hemorrhages" or "cerebral hemorrhage" or "hematencephalon" or "encephalorrhagia").mp. [mp=title, abstract, heading word, drug trade name, original title, device manufacturer, drug manufacturer, device trade name, keyword, floating subheading word, candidate term word]

7 ("randomized controlled trial" or "controlled clinical trial" or "clinical trial" or "randomized" or clinical trial or "placebo" or "randomly" or "cross-over studies").mp. [mp=title, abstract, heading word, drug trade name, original title, device manufacturer, drug manufacturer, device trade name, keyword, floating subheading word, candidate term word]

8 1 or 4

9 2 or 5

10 3 or 6

11 7 and 8 and 9 and 10

Cochrane central library

#1 MeSH descriptor: [Cerebral Hemorrhage] explode all trees
#2 MeSH descriptor: [Brain Ischemia] explode all trees
#3 MeSH descriptor: [Stroke] explode all trees
#4 ("cerebrovascular accident*" OR Stroke* OR "Cerebrovascular Apoplexy" OR "Brain Vascular Accident" OR "Brain Vascular Accidents" OR Apoplexy OR "Cerebral Stroke" OR "Cerebral Strokes" OR "Ischemic Encephalopathy" OR "Ischemic Encephalopathies" OR "Cerebral Ischemia" OR "Cerebral Ischemias" OR "Cerebral Hemorrhage" OR "Cerebrum Hemorrhage" OR "Cerebrum Hemorrhages" OR "Cerebral Parenchymal Hemorrhage" OR "Cerebral Parenchymal Hemorrhages" OR "Intracerebral Hemorrhage" OR "Intracerebral Hemorrhages" OR "Cerebral Hemorrhages" OR "Cerebral Brain Hemorrhage" OR "Cerebral Brain Hemorrhages"):ti,ab,kw
#5 #1 OR #2 OR #3 OR #4
#6 MeSH descriptor: [Brain-Computer Interfaces] explode all trees
#7 ("Brain Computer Interfaces" OR "Brain-Computer Interface" OR "Brain Computer Interface" OR "Brain-Machine Interfaces" OR "Brain-Machine Interface" OR "Brain Machine Interface" OR "Brain Machine Interfaces"):ti,ab,kw
#8 #6 OR #7
#9 MeSH descriptor: [Electroencephalography] explode all trees
#10 ("EEG" OR "Electroencephalogram" OR "Electroencephalograms"):ti,ab,kw
#11 #9 OR #10
#12 #5 AND #8 AND #11 in Trials
